# Idiosyncratic Biogenesis of Intracellular Pathogens-Containing Vacuoles

**DOI:** 10.3389/fcimb.2021.722433

**Published:** 2021-11-11

**Authors:** Bethany Vaughn, Yousef Abu Kwaik

**Affiliations:** ^1^ Department of Microbiology and Immunology, University of Louisville, Louisville, KY, United States; ^2^ Center for Predictive Medicine, College of Medicine, University of Louisville, Louisville, KY, United States

**Keywords:** vacuoles, intracellular pathogens, biogenesis, lysosomes, trafficking

## Abstract

While most bacterial species taken up by macrophages are degraded through processing of the bacteria-containing vacuole through the endosomal-lysosomal degradation pathway, intravacuolar pathogens have evolved to evade degradation through the endosomal-lysosomal pathway. All intra-vacuolar pathogens possess specialized secretion systems (T3SS-T7SS) that inject effector proteins into the host cell cytosol to modulate myriad of host cell processes and remodel their vacuoles into proliferative niches. Although intravacuolar pathogens utilize similar secretion systems to interfere with their vacuole biogenesis, each pathogen has evolved a unique toolbox of protein effectors injected into the host cell to interact with, and modulate, distinct host cell targets. Thus, intravacuolar pathogens have evolved clear idiosyncrasies in their interference with their vacuole biogenesis to generate a unique intravacuolar niche suitable for their own proliferation. While there has been a quantum leap in our knowledge of modulation of phagosome biogenesis by intravacuolar pathogens, the detailed biochemical and cellular processes affected remain to be deciphered. Here we discuss how the intravacuolar bacterial pathogens *Salmonella, Chlamydia, Mycobacteria, Legionella, Brucella, Coxiella*, and *Anaplasma* utilize their unique set of effectors injected into the host cell to interfere with endocytic, exocytic, and ER-to-Golgi vesicle traffic. However, *Coxiella* is the main exception for a bacterial pathogen that proliferates within the hydrolytic lysosomal compartment, but its T4SS is essential for adaptation and proliferation within the lysosomal-like vacuole.

## Introduction

Immune cells are equipped with a variety of receptors that recognize foreign material and particles permitting them to internalize the particles into a plasma-membrane derived vacuole that follows a maturation process that ultimately yields a phagolysosome (Vieira et al., 2002). Additionally, non-immune cells are equipped with receptors utilized by bacterial pathogens such as *Chlamydia* to enter by receptor-mediated endocytosis (Hybiske and Stephens, 2007a). Phagocytosis is a form of endocytosis that refers to cellular uptake of large particles and is initiated by the interaction of surface receptors with their cognate ligand (Vieira et al., 2002; Kinchen and Ravichandran, 2008). Upon phagocytosis, the nascent phagosome undergoes a complex sequence of maturation events governed by regulators of the endosomal-lysosomal degradation pathway that yields an acidic degradative compartment designated as the phagolysosome (Pitt et al., 1992; Vieira et al., 2002; Rama et al., 2015). Therefore, successful evolution of intra-vacuolar pathogens is dependent on their capacity to evade innate immune pathways and modulate biogenesis of their vacuole and adaptation to the unique micro-environment within the vacuole (Mnich et al., 2020). Depending on the phagocytosed bacterial pathogen, the default maturation of the phagosome along the endosomal-lysosomal pathway is overridden by specific pathogenic factors that interfere with the fate of the pathogen-containing vacuole (Do et al., 2016). The interference and divergence of the pathogen-containing vacuole from the default endosomal-lysosomal pathway is at the crux of the successful evolution of many intra-vacuolar pathogens to adapt to the intra-vacuolar microenvironment and inflict pathology and disease (Çakır et al., 2020; Leseigneur et al., 2020; Sachdeva and Sundaramurthy, 2020). Despite being an accidental human pathogen *Legionella* has evolved similar lysosomal evasion mechanisms to avoid killing by its natural amoeba hosts and human macrophages.

Intra-vacuolar bacterial pathogens have evolved to manipulate interactions between the pathogen-containing vacuole and endocytic vesicles to interfere with the progressive transfer of endo/lysosomal membrane and luminal constituents to the pathogen-containing vacuole (Vieira et al., 2002; Mitchell et al., 2016). There are several important regulators that govern endosomal maturation that have been identified including the Rab5, Rab7, and Rab 9 GTPases (Rama et al., 2015; Kucera et al., 2016; Szulc-Dabrowska et al., 2020). Pathogens also undergo various aspects of gene regulation to adapt to the intracellular environment (Chakravarty and Massé, 2019; Leipheimer et al., 2019).

**Figure 1:**
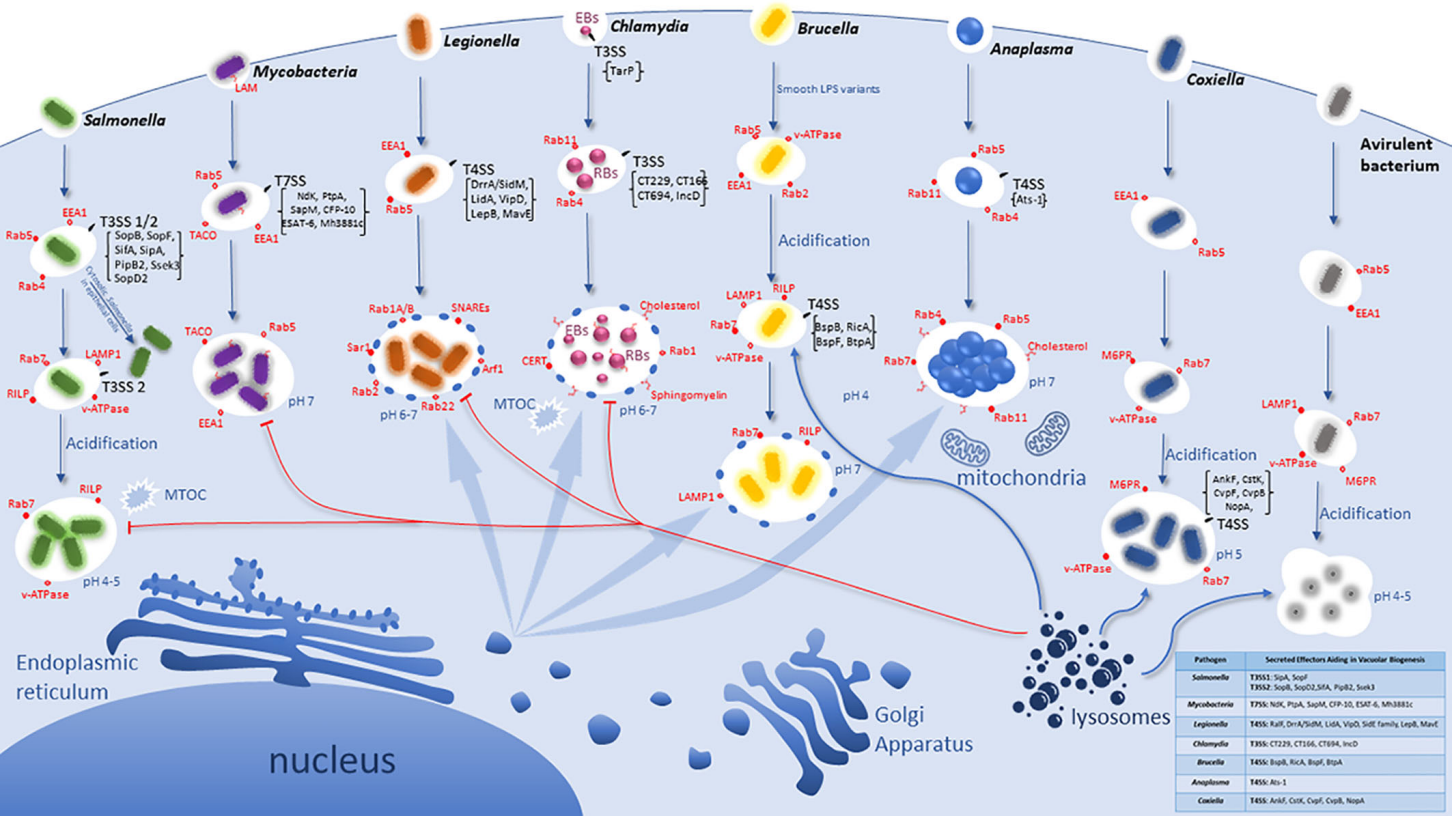
Idiosyncratic Biogenesis of the Vacuoles harboring Intracellular bacterial Pathogens. Pathogens have evolved distinct pathogenic factors and biochemical mechanisms to evade degradation upon entering host cells and enable their proliferation within modified vacuoles. All the listed pathogens have evolved specialized secretion machineries (Type 3 - Type 7) that inject effector proteins into the host cytosol to interact and modulate specific host cell proteins and/or processes. However, the T4SS of Coxiella is not functional until vacuolar acidification and is specifically used in replication but may not be involved in vacuole biogenesis. These seven intra-vacuolar bacterial pathogens, *Salmonella, Mycobacteria, Legionella, Chlamydia, Brucella, Anaplasma*, and *Coxiella*, interfere with endocytic, exocytic and/or ER-to-Golgi vesicle trafficking to evade lysosomal degradation. *Salmonella* and *Coxiella* proliferate within an acidified late endosome/lysosomal-like vacuole whereas *Legionella, Chlamydia*, *Anaplasma* as well as *Brucella* intercept and modulate ER-to Golgi vesicles for their vacuole biogenesis.

Bacterial pathogens that have adopted an intracellular lifestyle have evolved different strategies to avoid trafficking to the degradative phagolysosome (Duclos et al., 2000). A distinct group of bacterial pathogens, such as *Listeria, Shigella*, and *Rickettsia*, escape from the phagosome to the cytosol (Ray et al., 2009; Curto et al., 2019a; Curto et al., 2019b; Green et al., 2020; Wang et al., 2020b). The cytosolic pathogens have evolved mechanisms to evade innate immune mechanisms of macrophages such as autophagy (Thomas et al., 2020), activation of the inflammasomes (Snaka and Fasel, 2020), and other host innate sensing of foreign material within the cytosol, and these are reviewed elsewhere (Kubelkova and Macela, 2019; Thakur et al., 2019). In contrast, we focus this review on the group of bacterial pathogens that reside and proliferate within a modified vacuole whose altered biogenesis is governed by the interaction of injected pathogenic effectors with cellular targets leading to its diversion from the default endosomal-lysosomal pathway. These intra-vacuolar pathogens have evolved to alter their initial phagosomal compartment in order to halt its maturation or divert it from the endocytic pathway to avoid the fatal fate of degradation within the phagolysosome (Garin et al., 2001). The intra-vacuolar niche within the host cell benefits intracellular pathogens as there is limited competition with other bacteria, the intracellular environment can provide vital nutrients, and the pathogen is no longer susceptible to complement or neutralizing antibodies (Casadevall, 2008; Casadevall and Pirofski, 2011). However, although most intra-vacuolar pathogens proliferate within a phagosome that is stalled from maturing into a phagolysosome, the pathogenic factors and biochemical mechanisms involved in this interference are all idiosyncratic and there is no common theme among intra-vacuolar pathogens in their evolution and interference with biogenesis of their replicative vacuoles (**Figure 1**).

The abundant series of steps, molecules, and checkpoints that must take place for proper endosomal maturation allow for many possible host targets to be interfered with and modulated by pathogens, and thus potential evolution and adaptation of the pathogen to exploit the endosomal pathway to the pathogen advantage (Rama et al., 2015). Intracellular bacteria have evolutionarily gained the capacity to subvert signaling and trafficking pathways by employing T3SS-T7SS (Weber et al., 2009; Green and Mecsas, 2016; Monjarás Feria and Valvano, 2020). These secretion systems are multiprotein complexes that span both bacterial membranes and inject a variety of effector proteins directly into the host cytoplasm *via* a molecular nano-syringe (Ninio and Roy, 2007; Schroeder and Hilbi, 2008; Steele-Mortimer, 2008; Weber et al., 2009; Green and Mecsas, 2016). Bacterial effector proteins modulate a myriad of host cell processes and play an essential role in mediating vesicle trafficking pathways controlled by Rab GTPases and V-ATPases involved in phagosome biogenesis (Weber et al., 2009; Rama et al., 2015). Each of the following Gram-negative pathogens has evolved to be equipped with a unique toolbox of a set of effectors that are injected into the host cell, with a myriad of effects on cell biology. Here, we focus on the intra-vacuolar pathogens *Salmonella*, *Mycobacteria, Legionella, Chlamydia, Brucella, Anaplasma*, and *Coxiella* to discuss their evolution and adaptation to modulate and exploit host endocytic and exocytic trafficking pathways to create an idiosyncratic vacuolar niche suitable for intra-vacuolar proliferation (**Figure 1**).

## The Late Endosome-Like Vacuole of *Salmonella enterica*

*Salmonella enterica* serovar Typhimurium is a major cause of food‐borne enterocolitis in humans and a systemic, typhoid-like disease in genetically susceptible mice (Tsolis et al., 1999; Brumell and Scidmore, 2007). These bacteria are facultative intracellular pathogens that initially interact with intestinal epithelial cells at the onset of infection primarily through the M cells of Peyer’s patches, however they infect a wide variety of cell types in their host during systemic infection (Gordon et al., 2008). *S. enterica* serovar Typhimurium invades and replicates within host cells using two different T3SS and interferes with the host cell cycle to enhance invasion (Galan and Wolf-Watz, 2006; Brumell and Scidmore, 2007; Mambu et al., 2020). The first injected group of effectors is delivered across the epithelial plasma membrane by the T3SS1 (Lou et al., 2019) to modulate host signal transduction pathways, including the activation of Rho family GTPases, to induce actin rearrangements that drive the ruffling of the cell surface, IAM formation (fluid-filled infection associated macropinosomes), and the uptake of the bacteria into a *Salmonella*-containing vacuole (SCV) (Schlumberger and Hardt, 2006; Ramos-Morales, 2012; Stevenin et al., 2019). This is an essential pathogenic adaptation to infect non-phagocytic cells, since the recognition of the T3SS by macrophages induces activation of the inflammasome, resulting in caspase-1-mediated pyroptosis (Brennan and Cookson, 2000; Wang et al., 2020a). The T3SS2 of *S. enterica* is expressed within the SCV in response to acidic pH and Mg^2+^ limitations (Ramos-Morales, 2012). The SCV is a pleomorphic organelle characterized by filamentous projections termed *Salmonella*-induced filaments (SIFs) (Garcia-del Portillo et al., 1993; Kumar and Valdivia, 2009). Some late endosome-lysosomes markers accumulate on the SCV and enable its traffic along microtubules to the microtubule-organizing center (Bakowski et al., 2008; Kumar and Valdivia, 2009; Gao et al., 2018). Thus, the modification of the SCV is governed by T3SS2-secreted effector proteins located on *Salmonella* pathogenicity island 2 (SPI-2), allowing the bacteria to sense the acidic vacuolar environment, modulate vesicle traffic, and ensue replication (Kenney, 2019).

SCV biogenesis can be divided into three stages (**Figure 1**): early (10 min–1 h post infection), intermediate (1 h–4 h), and late (>4 h) (Ramos-Morales, 2012). Initially in epithelial cells, the SCV interacts with early endosomes and acquires the markers Rab4, Rab5, and EEA1 (Steele-Mortimer et al., 1999; Smith et al., 2005). Overexpression of Rab5 causes the retention of the early endosomal marker EEA1 and promotes homotypic early endosome fusion (Baldeon et al., 2001; Brumell and Scidmore, 2007). Approximately 15 to 60 minutes post infection, lysosomal glycoprotein (Lgp) LAMP-1 is recruited to the SCV membrane. The SCVs are thought to acquire Lgp by interacting with a Rab7-positive/Lgp-positive/manose-6-phosphate (M6PR)-negative/Cathepsin D-negative vesicles (Meresse et al., 1999). During this stage of SCV maturation, active Rab7 recruits its effector RILP and promotes centripetal SCV movement to the microtubule organizing center (Harrison et al., 2004). This is characteristic of the intermediate state in SCV development (Ramos-Morales, 2012). The vATPase is acquired by the SCV leading to its acidification with a pH of 4.0-5.0, which is required for the expression of various pathogen genes required for adaptation and proliferation within the SCV (Buchmeier and Heffron, 1991; Mills and Finlay, 1998; Garvis et al., 2001; Cuellar-Mata et al., 2002; Brumell and Scidmore, 2007; Smith et al., 2007; Lahiri et al., 2010).

During the late stage of SCV maturation a set of T3SS effectors generate different types of tubular perinuclear networks, SIFs, *Salmonella*-induced secretory carrier membrane protein 3 (SCAMP3) tubules (SISTs), and LAMP1-negative tubules (LNTs) (Ramos-Morales, 2012). However, because SCVs do not fuse with lysosomes, it is suggested that *S. enterica* can modify the Rab network associated with phagosome maturation and the Lgp acquisition that contributes to the control of SCV during infection is promoted by the positioning of Rab7 (Brumell and Scidmore, 2007). Additionally, the SopB translocated effector manipulates the SCV surface charge by dissociating several host-cell endocytic trafficking proteins from the SCV: inhibiting lysosomal fusion (Bakowski et al., 2010; Kaur and Jain, 2012) (**Figure 1**). SopB has also been found to promote IAM formation and also trigger the formation of SVATs (spacious vacuole-associated tubules), resulting in the shrinkage of the vacuole (Stevenin et al., 2019). To evade the innate immune response of macrophages, the injected SopF effector is involved in blocking autophagy (Xu et al., 2019).

Upon its maturation, the SCV becomes a replicative niche for *Salmonella*, although there are a small number of WT cytosolic bacteria that routinely escape from the SCV, *Salmonella*-induced filament A (*sifA*) mutants readily escape into the cytoplasm (Beuzon et al., 2000; Kumar and Valdivia, 2009). This indicates a role for SifA in maintaining integrity for the SCV and SifA is likely to play a role in systemic disease as *sifA* mutants fail to establish infection in mice (Stein et al., 1996; Kumar and Valdivia, 2009; Liss et al., 2017). *Salmonella* injects the SopD2 effector, which modulates Rab7 activity leading to interference with endosome-to-lysosome trafficking (D'Costa et al., 2015). In addition to SCV escape in epithelial cells, a subset of SCVs follow retrograde vesicle traffic from the Golgi back to the endoplasmic reticulum, which is also dependent on the T3SS2 effector PipB2 associated with a decrease in the T3SS1 effector SipA (Ramos-Morales, 2012). Kinesin is involved in maintaining the structure and position of the Golgi apparatus along with transport of vesicles (Goldstein and Philp, 1999; Allan et al., 2002). Work performed by Boucrot et al. determined SifA targeted SKIP, a host protein that down-regulates the recruitment of kinesin on the SCV (Boucrot et al., 2005). Recently, the SifA/SKIP complex has been shown to be necessary for the activation of the kinesin-1 recruited by PipB2, revealing a functional interaction important for formation of tubules in membrane exchange and nutrient supply (Alberdi et al., 2020). The SseK3 effector targets various host small GTPases (Gan et al., 2020).

In epithelial cells, roughly 10% of intra-vacuolar bacteria can escape the SCV and reside in the host cytoplasm, where the bacteria can proliferate at high rates to reach a progeny of >100 cfu’s per infected cell (Kumar and Valdivia, 2009; Knodler, 2015; Castanheira and Garcia-Del Portillo, 2017). Interestingly, *S. Typhimurium* does not escape into the cytosol in fibroblasts, allowing endosomal and lysosomal membranes to accumulate around the SCV forming large aggregates that engage the autophagy machinery in an ATG9A-independent manner (Kageyama et al., 2011; Lopez-Montero et al., 2016). Furthermore, a recent study concluded Rab7-positive vesicles are more acidic as compared to the Arl8b-positive vesicles (Johnson et al., 2016). Arl8b, a small lysosomal GTPase, promotes membrane localization of Vps41, a HOmotypic fusion and Protein Sorting complex (HOPS). This may suggest that *Salmonella* recruits late endosomal and lysosomal host membrane proteins for access to host nutrients to ensure its intracellular survival and replication (Sindhwani et al., 2017). More recently, Eswarappa et al. concluded that a majority of SCVs only have one bacterium per vacuole, ultimately increasing the vacuolar load per cell, the host would have to target each SCV separately with lysosomes and other microbicidal agents potentially affecting the susceptibility of the host cell to other pathogens (Eswarappa et al., 2010). Consistent with the IAM and/or SVAT formation causing the SCV to undergo size modifications, the number of bacteria present in the SCV causes vacuole size increase and decrease.

Both SCV escape and retrograde movement of the SCV have been associated with the ability of *Salmonella* to achieve cell-to-cell transfer in order to repeat the intracellular cycle (Knodler et al., 2010; Ramos-Morales, 2012). Thus, *Salmonella* has evolved multiple mechanisms for adaptation and survival within an idiosyncratic acidified late endosome-like vacuole. Ultimately, its successful pathogenic evolution and adaptation to the intra-vacuolar microenvironment is dependent of its ability of membrane remodeling, interactions with the endosomal/lysosomal pathway, actin rearrangements and microtubule-based movement.

## The Early Endosome-Like Vacuole of *Mycobacterium tuberculosis*

*Mycobacterium tuberculosis* is the causative agent of Tuberculosis, with an estimated 10 million infected last year (Clemens and Horwitz, 1995; Kumar and Valdivia, 2009). While the majority of infected people are asymptomatic, 1-2 million people die annually of the disease. The slow-growing and chronic nature of the infection and the difficulty in adherence to the long-term therapy by patients has potentially contributed to the rise of hyper-virulent multidrug-resistant strains (Clemens and Horwitz, 1995; Kumar and Valdivia, 2009; Matteelli et al., 2014). *M. tuberculosis* in most cases induces irreversible necrosis of lung tissue as a result of exacerbated inflammation and enhanced recruitment of myeloid cells with progression to chronic disease associated with a reduction in inflammatory mediators (Almeida et al., 2017).

*M. tuberculosis* is a highly aerobic bacteria that primarily infects macrophages and monocytes (Clemens and Horwitz, 1995). However, outside the lungs *M. tuberculosis* can disseminate to any organ *via* the lymphatic system (Bussi and Gutierrez, 2019; Moule and Cirillo, 2020). Infected alveolar macrophages elicit recruitment of more monocytes to the infection site creating a granuloma within the infected lung to be the sites of chronic inflammation (Arora et al., 2020) and a growing niche for replication where the pathogen utilize lipids for nutrition (Cambier et al., 2014; Warsinske et al., 2017; Del Portillo et al., 2019; Maurya et al., 2019) and targets host mitochondria to trigger metabolic changes (Mohareer et al., 2020).

A prominent mannose-containing lipoglycan of the *M. tuberculosis* cell wall, the terminal mannose-capped lipoarabinomannan (ManLAM) is a high molecular mass amphipathic lipoglycan that plays a crucial role in mycobacterial survival. ManLAM facilitates *M. tuberculosis* entry into phagocytes, regulates the intracellular trafficking network as well as the immune responses of infected host cells (Kang et al., 2005; Turner and Torrelles, 2018). After entry into macrophages, *M. tuberculosis* resides in a vacuole that is arrested from maturation at the early endosomal stage where early endosomal markers (EEA1, Rab5) accumulate on the pathogen-containing vacuole (**Figure 1**) (Clemens and Horwitz, 1995; Kumar and Valdivia, 2009). The *M. tuberculosis*-containing vacuole has a near-neutral pH and retains its ability to interact with early endosomes. In addition to ManLAM, it is suggested that the *M. tuberculosis* glycosylated phosphatidylinositol lipoarabinomannan (LAM) excludes V-ATPase from the vacuole and limits the induction of host autophagy (Vergne et al., 2004; Augenstreich and Briken, 2020). Ferrari et al. has reported that 90% of *M. tuberculosis* vacuoles retain Tryptophan Aspartate- Containing Coat protein/Coronin 1 (TACO) and macrophages incubated with dead bacteria shortly release TACO, suggesting long-term retention of TACO on the *Mycobacteria* containing vacuole (MCV) contribute to inhibition of phagosomal maturation (Ferrari et al., 1999).

In addition to the role of LAM and ManLAM, *M. tuberculosis* injects protein effectors through its ESX T7SS (Guo et al., 2019), to maintain the early endosomal characteristics of the vacuole (Crosskey et al., 2020) and modulate autophagosome formation (Garg et al., 2020). Mutants of *Mycobacteria* that lack the ESX-1 secretion system are less efficient in surviving inside macrophages (MacGurn and Cox, 2007). Phosphoinositides are low-abundance lipid constituents on eukaryotic membranes that determine vesicle trafficking/organelle identity within eukaryotic cells (Di Paolo and De Camilli, 2006; Sasaki et al., 2009). *Mycobacteria* are able to disrupt the host cell phosphoinositide pattern and other processes through secretory phosphatases (Koliwer-Brandl et al., 2019). ManLAM co-operates with the secreted acid phosphatase SapM to block phagosome fusion with late endosomes by inhibiting PI3P deposition on the phagosome surface (Deretic et al., 2006; Wong et al., 2018). Specifically, SapM inhibits PI3P deposition on the phagosome surface *via* PI3P hydrolysis, contributing to inhibition of phagosomal maturation (Vergne et al., 2005) The PtpA T7SS-injected effector protein is a low molecular weight tyrosine phosphatase (Cowley et al., 2002) essential for growth in human macrophages (Bach et al., 2008). PtpA binds to the host H subunit of V-ATPase rendering it unable to bind to the *M. tuberculosis* containing vacuole and impedes acidification of the *M. tuberculosis*-containing vacuole (Wong et al., 2011; Saha et al., 2020). PtpA colocalizes with a regulator of membrane fusion, VPS33B, in the host cytosol, resulting in dephosphorylation of the VPS33B subunit of the Homotypic Fusion and Vacuole Protein Sorting (HOPS) complex. In infected macrophages, this process prevents anchoring of the HOPS complex to SNARE molecules (Bach et al., 2008). PtpA interaction with host V-ATPase is required for the dephosphorylation of VPS33B and subsequent exclusion of V-ATPase from the phagosome, inhibiting phagosome-lysosome fusion during *M. tuberculosis* infection (Wong et al., 2011). Protein kinase A (PtkA), encoded on the same operon as PtpA, increases PtpA activity *via* phosphorylation, thereby enhancing PtpA phosphatase activity (Zhou et al., 2015; Wong et al., 2018). The NdK effector, a nucleoside diphosphate kinase, dephosphorylates cellular Rab7-GTP and Rab5-GTP (Sun et al., 2010). This prevents “Rab switching” and Rab7- dependent fusion of the vacuole which halts endosomal maturation from lysosomal fusion. The eukaryotic-type protein kinase G (PknG) is a member of the STPK family in *M. tuberculosis* and is associated with mycobacterial survival within macrophages, metabolic regulation, and inhibits phagosome lysosome fusion (Av-Gay and Everett, 2000; Cowley et al., 2004; Scherr et al., 2007; O'Hare et al., 2008; Ge et al., 2021). PknG inhibits autophagosome maturation by phosphorylating TBC1D4/AS160 (TBC1 domain family member 4) to suppress its GTPase-activating protein (GAP) activity toward RAB14 and also by binding to host small GTPase RAB14 to block RAB14-GTP hydrolysis (Ge et al., 2021).

Recently, *M. tuberculosis* has been found to rapidly escape from phagosomes within infected macrophages *via* the activation of host cytosolic phospholipase A_2_ and establish residence in the cytoplasm of the host cell (Jamwal et al., 2016). Individual *M. tuberculosis* strains display a strain-specific capacity to escape from phagosomes, with the predominant population of JAL2287, JAL2549, and MYC431 bacteria strains localizing to the cytosol (Jamwal et al., 2016). Previously, a related species *Mycobacterium marinum*, has shown to escape phagosomes and can recruit host cell cytoskeletal factors to induce actin polymerization leading to direct cell to cell infection (Stamm et al., 2003). Also, virulent strains of *M. tuberculosis* have been shown to translocate from the phagosome into the cytosol of dendritic cells by interfering with the TLR-2-MyD88 signaling pathway (Rahman et al., 2014). The effectors PtpA and SapM are also translocated *M. marinum* phosphatases, which dephosphorylate phosphatidylinositol-3-phosphate (PtdIns3P) on the cytoplasmic side of MCV, modulating the MCV PI pattern (Koliwer-Brandl et al., 2019). PtdIns3P is required for phagolysosome progression along the endosomal pathway and a reduction of PtdIns3P on MCV likely impairs lysosomal fusion thus promoting intracellular replication of *M. marinum* and escape into the cytoplasm (Murray et al., 2002; Koliwer-Brandl et al., 2019). Additionally, Mh3881c, an ESX-1 protein, has been found to co-secrete with CFP-10/ESAT-6 and this co-dependent secretion is required for *M. marium* intracellular growth in macrophages and correlates with its role in inhibiting phagosome maturation (Xu et al., 2007). This shows plasticity in the adaptation of *M. tuberculosis* and *M. marinum* for survival and growth in susceptible hosts. Thus, *M. tuberculosis* have evolved to arrest phagosome maturation at an early stage of biogenesis through exclusion of the V-ATPase and directly alters various host signaling pathways through the secretion of effector proteins that alter phagosomal maturation to generate an idiosyncratic early endosome-like replicative niche (Bussi and Gutierrez, 2019).

## The ER-Derived Vacuole of *Legionella pneumophila*

*Legionella pneumophila* is a Gram-negative, aquatic, facultative intracellular bacterium that is the causative agent of Legionnaires’ disease (Fraser et al., 1977; McDade et al., 1977). It naturally infects a wide variety of freshwater amoebas and other protists, but the organism can also accidentally infect humans upon the inhalation of contaminated aerosols (Albert-Weissenberger et al., 2007; Best and Abu Kwaik, 2018). Once inhaled, the pathogen is able to invade and replicate in alveolar macrophages (Aridor et al., 1995; Albert-Weissenberger et al., 2007).

The Dot/Icm-encoded T4SS of *L. pneumophila* delivers effector proteins into the mammals and protists hosts (Segal et al., 2005). The Dot/Icm-encoded T4SS of *L. pneumophila* injects ~350 effector proteins into the host cytosol to target specific host processes and pathways in order to modulate biogenesis of the *Legionella*-containing vacuole (LCV) (Roy et al., 1998; Coers et al., 1999; Segal et al., 2005; Li et al., 2020). Within alveolar macrophages and the amoeba hosts, the LCV evades fusion with vesicles of the endosome-lysosomal pathway (Swanson and Isberg, 1995), and intercepts ER-to-Golgi vesicle traffic to become an ER-derived vacuole associated with polyubiquitinated proteins (**Figure 1**) (Horwitz, 1983; Swanson and Isberg, 1995; Kitao et al., 2020). The ER-to-Golgi vesicle traffic is regulated by Rab1A/Rab1B (Nuoffer et al., 1994; Wilson et al., 1994), Rab2 (Tisdale et al., 1992), and at least one SNARE complex composed of one v-SNARE (Sec22b) and three cognate t-SNAREs (syntaxin 5, membrin, and Bet1) (Hay et al., 1997; Xu et al., 2000). Both Rab1 and Arf1 have been found to be localized to the LCV by 30 min post infection (Nagai et al., 2002; Derre and Isberg, 2004; Kagan et al., 2004). In addition, Sar1 and Arf1, two small GTPases, regulate the formation of COPII and COPI-coated vesicles and are required for the production of early secretory vesicles (Aridor et al., 1995; Scales et al., 1997; Duden, 2003). The expression of dominant interfering mutants in the three small GTPases (Sar1, Rab1, and Arf1) inhibits the formation of LCV and decreases intracellular survival of the organism (Derre and Isberg, 2004; Kagan et al., 2004; Robinson and Roy, 2006).

The function of many Dot/Icm-injected effectors is still to be determined. However, among the characterized effectors involved in the LCV biogenesis, RalF functions as a GEF to recruit the ADP-ribosylation factor 1 (Arf1) to the LCV (Nagai et al., 2002). This enables the GEF-like RalF effector to modulate membrane transport in the secretory pathway (Alix et al., 2012). Additionally, the DrrA/SidM effector functions to recruit and regulate Rab1 activity at the LCV. DrrA/SidM preferentially recruits Rab1 and tethers ER-derived vesicles to the LCV, mediated by another *L. pneumophila* effector, LidA (Ninio and Roy, 2007). DrrA/SidM may compete with endogenous guanine nucleotide exchange factors (GEFs) to redirect Rab1 from its normal secretory intracellular localization to plasma membrane-derived vesicles (Murata et al., 2006). The LepB effector accumulates on the LCV as DrrA/SidM and Rab1 cycle off and has been shown to function as a GTPase activating protein (GAP) for Rab1 (Ingmundson et al., 2007; Mishra et al., 2013). While LidA binds several Rab GTPases including Rab1, it may sequester Rab proteins or tether ER-derived vesicles with the LCV to facilitate SNARE-mediated fusion (Mishra et al., 2013). The VipD effector of *L. pneumophila* belongs to a family of bacterial effectors that contain the N-terminal lipase domain and a C-terminal domain (Ku et al., 2012). VipD localizes to early endosomes *via* the C-terminal domain and interferes with endosomal trafficking through blocking interaction with Rab5 and Rab22 with EEA1 (Ku et al., 2012). VipD binds to the endosomal regulator Rab5 and triggers the hydrolytic phospholipase A_1_ activity in VipD, causing the removal of the lipid phosphatidylinositol 3-phosphate facilitating endosomal lysosomal avoidance by *L. pneumophilia* (Gaspar and Machner, 2014). The SidE family of effector proteins (SidE, SdeA, SdeB, and SdeC) have been found to catalyze the non-canonical ubiquitination of Rab small GTPases, leading to a potential role in vesicular trafficking (Wang et al., 2018).

Recently, the MavE translocated effector has been shown to be indispensable for diverting the LCV from the endosomal-lysosomal pathway and is essential for intracellular replication in human macrophages and amoeba (Vaughn et al., 2021). MavE has an interaction with another effector protein, a metaeffector, YlfA/LegC7 that along with two other effectors (LegC2 & LegC3), assembles in a complex on the LCV and interacts with ER-derived vesicles to initiate membrane fusion (Jahn and Scheller, 2006; Huang et al., 2011; O'Brien et al., 2015; Shi et al., 2016; Urbanus et al., 2016). Importantly, the crystal structure of MavE shows a eukaryotic NPxY motif that interacts with phosphotyrosine-containing proteins, and this motif is essential for the function of MavE in lysosomal evasion (Vaughn et al., 2021). However, the role of the LegC7/LegC2/LegC3 complex and its potential interaction with MavE in the biological functions of MavE is still to be determined.

A key virulence determinant of *L. pneumophila* is the ability of the LCV to be remodeled into an ER-derived vacuole diverted from traffic through the endosomal-lysosomal pathway (Isberg et al., 2009; Richards et al., 2013; Kotewicz et al., 2017). While the mechanisms *L. pneumophilia* employs to evade lysosomal fusion is still unclear, the T4SS effector proteins DrrA/SidM, LidA, VipD, LepB and MavE have been shown to play roles in diversion of the LCV from the endosomal-lysosomal pathway. The long-term evolution of *L. pneumophila* with various amoeba as the natural hosts, and inter-kingdom as well as inter-bacterial horizontal gene transfer has most likely shaped the evolution of the pathogen to adapt to the intra-vacuolar environment of eukaryotic cells.

## Intercepting the Exocytic Pathway by *Chlamydia trachomatis*

*Chlamydia trachomatis* is an obligate, Gram-negative, intracellular bacteria that is responsible for the most reported sexually transmitted bacterial infection in the United States. In developing countries, it is known to cause a blinding disease known as trachoma. The pathogen replicates in the host cell in a vacuole termed the *Chlamydia* inclusion (van Ooij et al., 1997; Johnson et al., 2014), and has a biphasic developmental intracellular life cycle (**Figure 1**) (Birkelund, 1992; Brumell and Scidmore, 2007; Kunz et al., 2019). The first phase is an infectious, extracellular form termed the elementary body (EB) that is metabolically inert but exhibiting metabolic activity under axenic conditions (Omsland et al., 2014). The second is an intracellular, non-infectious, and metabolically active reticulate body (RB) (Moulder, 1991). To induce uptake into host cells, the EB utilizes a T3SS to inject an effector designated as translocated actin-recruiting phosphoprotein (TARP) across the host cell membrane (Clifton et al., 2004; Clifton et al., 2005; Jewett et al., 2006). TARP remodels the host actin at the bacterial entry site and activates a master regulator of lamellipodium formation (Rac1) (Marei and Malliri, 2017; Tolchard et al., 2018). The EB then exploits the host cytoskeleton to facilitate entry into the host cell and form the inclusion (Hybiske and Stephens, 2007b). Within the inclusion, the EBs rapidly differentiate into RBs and the pathogen begins to replicate by binary fission within the inclusion (**Figure 1**) (Hybiske and Stephens, 2007b). The infection cycle lasts 48 -72 hours, when the RBs then differentiate back into infectious EBs and are released from the host cell by extrusion or lysis (Hybiske and Stephens, 2007b). While *Chlamydiae* and *chlamydiae*-related organisms share a strictly intracellular biphasic developmental cycle, depending on the species, the cycle can last up to 10 days (Derre, 2015). This review focuses on intracellular trafficking of *C. trachomatis* and the establishment of other *Chlamydia* species to manipulate the host cellular environment has been shown to have effector-dependent subversion from the endocytic pathway, interaction with the mitochondria and/or the ER (Croxatto and Greub, 2010; Collingro et al., 2011; Mehlitz et al., 2014).

Early in the infection cycle, *Chlamydia trachomatis* relies on the modification of the inclusion membrane to disguise the vacuole as a host organelle that is separate from the endocytic pathway. The bacteria actively modify the inclusion by inserting bacterial inclusion effector proteins (Incs) and host lipids are acquired into its membrane early in infection (Rzomp et al., 2003; Hybiske and Stephens, 2007b). Inc are commonly thought to interact with host cell components to facilitate the organisms’ growth, survival and subversion throughout the host cell (Gauliard et al., 2015). The *Chlamydia* inclusion recruits Rab GTPases to the inclusion (Rzomp et al., 2003), and rapidly exits the endocytic pathway and associates with Rabs 1, 4 and 11 (Rzomp et al., 2003). A few chlamydial Incs effectors have been shown to bind Rabs and specifically, Inc CT229 interacts directly with Rab4-GTP (Rzomp et al., 2006). The association with specific Rabs and the inclusion membrane helps identify the bacterial vacuole apart from the endocytic pathway.

Interestingly, *Chlamydia* also diverts its inclusion from the endocytic pathway by employing the host’s microtubule network for the duration of its infectious cycle (Mital et al., 2010). *Chlamydia* expresses two potential effectors that regulate actin depolymerization, CT166- an inhibitor of Rac1 and CT694- results in AHNAK- dependent loss of actin stress-fibers (Belland et al., 2001; Hower et al., 2009; Thalmann et al., 2010). The inclusion is trafficked to the peri-Golgi region of the host cell, maintaining close association with the microtubule organizing center (MTOC) (Brumell and Scidmore, 2007; Kumar and Valdivia, 2008; Mital et al., 2010). While the actin cytoskeleton may provide a protective framework for the inclusion, the lipid content of the inclusion is thought to help to identify it as an organelle separate from the endocytic pathway (Elwell and Engel, 2012). *Chlamydia* intercepts sphingomyelin (SM) trafficking from the Golgi to the plasma membrane and from basolateral vesicle traffic and incorporates it not only into the inclusion membrane but its own bacterial membrane (Moore et al., 2008). *Chlamydia* has also been shown to exploit the host protein CERT which regulates ceramide traffic from the ER to the Golgi (Derre et al., 2011; Elwell et al., 2011). CERT binds to the IncD effector to regulate and acquire SM incorporation into the inclusion (Derre et al., 2011; Elwell et al., 2011; Olson et al., 2019). Additional host lipids including lipids of the Golgi apparatus have been detected in the inclusion membrane including cholesterol, phosphatidic acid, and PtdIns(4)P (Carabeo et al., 2003; Beatty, 2008; Moorhead et al., 2010). *Chlamydiae* likely acquire cholesterol from the host cells as the pathogen lacks the enzymatic capacity for the lipid’s synthesis (Carabeo et al., 2003). Cholesterol and sphingomyelin are intercepted from the Golgi-derived vesicles and rerouted to the inclusion (Walpole et al., 2018). Cholesterol containing domains of the inclusion aid in recruiting Src-family kinases that also help maintain close association of the inclusion with the MTOC (Walpole et al., 2018). Ultimately, both cholesterol and sphingomyelin are important for the pathogen nutrition and growth but may also contribute to fusion of the cholesterol containing inclusion by “organelle mimicry” (Walpole et al., 2018).

Thus, the *Chlamydia* inclusion is modified through the secretion of *chlamydia*-specific effectors and is diverted from the endocytic pathway, and intercepts vesicles along the exocytic pathway to incorporate lipids and cholesterol into the inclusion (Brumell and Scidmore, 2007; Walpole et al., 2018). The development and dissemination of *C. trachomatis* is dependent upon the delivery of an arsenal of effector proteins that interfere with a wide diversity of host cell processes that influence vesicular trafficking, allowing the pathogen to establish an intracellular niche (Elwell et al., 2016; Bugalhao and Mota, 2019).

## Trafficking of *Brucella*-Containing Vacuole Along the Endosomal Pathway

*Brucella* are Gram-negative zoonotic pathogens known to infect a wide range of host species, but the three most common incidentally infecting humans are *Brucella melitensis* (primarily associated with goats and sheep), *Brucella suis* (primarily infecting swine) and *Brucella abortus* (primarily infecting cattle), (Boschiroli et al., 2002b; Xavier et al., 2010). *Brucella* is highly infectious, only requiring 10-100 bacteria to establish infection *via* inhalation (Hoover, 1997; Bossi et al., 2004) however, it can also be transmitted by consuming undercooked meat or unpasteurized dairy products (Bossi et al., 2004). Human infection with *Brucella* can result in a debilitating and chronic illness manifested as undulant fever (Young, 200; Roop et al., 2004). *Brucella* passes through the mucus membranes to infect phagocytic cells and when left untreated can disseminate throughout the body’s tissues and organs (Fernandez-Prada et al., 2003; Paixao et al., 2009).

Within macrophages, *Brucella* is trafficked within a vacuole through the endocytic pathway and this *Brucella* containing vacuole (BCV) is remodeled by the ER. The smooth LPS of *Brucella* with a O-side chain is crucial for entry into host cells, for vacuole biogenesis and for adaptation to the acidic pH and tolerance of the reactive oxygen intermediates (Roop et al., 2004; Haag et al., 2010). Rough LPS mutants of *Brucella* that lack O-side chain enter cells independently of lipid rafts and are rapidly killed by macrophages (Porte et al., 2003; Rittig et al., 2003; Pei and Ficht, 2004). However, less than 10% of internalized *Brucella* survive to establish a replicative BCV (He et al., 2006; von Bargen et al., 2012).

Once *Brucella* is internalized, the BCV traffics along the endocytic pathway, acquiring both early and late endosomal markers (**Figure 1**) (Pizarro-Cerda et al., 1998; Starr et al., 2012). Endosomal trafficking of the BCV proceeds with the acquisition of EEA1 and Rab5 (Pizarro-Cerda et al., 1998; Celli et al., 2003), these early endosomal markers are rapidly exchanged for the late endosomal markers Rab7, RILP, LAMP1, and vATPase. These events begin the conversion of the BCV into a more acidic compartment, which is essential for pathogenesis of this organism (Porte et al., 1999). The pH of the BCVs decreases to 4.0 within 1hr of bacterial entry and this pH persists for at least 5 hrs following infection (Porte et al., 1999). Importantly, the acidic pH seems to be involved in bacterial adaptation to the BCV, since inhibition of the vATPase results in a decreased bacterial viability (Porte et al., 1999).

However, the limited lysosomal fusion of the BCV is well controlled by the bacteria that have developed adaptations to resist degradation within the lysosomal microenvironment (Starr et al., 2008; Starr et al., 2012). The acidic pH micro-environment in the BCV is an activation signal for virulence genes as it activates the expression of the VirB T4SS that directs diversion of the BCV from the endocytic pathway into an ER-associated BCV (**Figure 1**) (Comerci et al., 2001; Boschiroli et al., 2002a; Celli et al., 2003). A critical virulence factor of *Brucella*, Cyclic-β-1,2-glucan, has a role in decreasing lysosomal fusion by depletion of BCV membrane cholesterol (Arellano-Reynoso et al., 2005). Since the BCV lumen is acidified and contains degradative enzymes, the pathogen mitigates the risk of degradation through expression of several proteins (HdeA and CydB) to resist the drop in pH, nitrosative damage, and antimicrobial peptides (Endley et al., 2001; Valderas et al., 2005; Roop et al., 2009). The *cydB* mutant strain has shown a diminished capacity for survival during stationary phase reflected by an inability to offset the effects of oxidative stress (Endley et al., 2001). The HdeA protein, which is conserved in several Gram-negative bacterial species, functions as chaperone protein in the periplasmic space to resist acid stress (Wu et al., 2008; Hong et al., 2012). The *Brucella* homolog of HdeA is expressed during stationary phase cultures (acidic conditions), which depends on the expression of the small RNA binding protein Hfq (HF-1), since deletion of the *hfq* gene significantly reduced levels of HdeA protein production (Valderas et al., 2005). HF-1 is required for entrance into stationary-phase physiology (Robertson and Roop, 1999). This post-transcriptional regulator of global gene expression promotes base pairing interactions of small RNAs with their target mRNAs to regulate target gene expression (Robertson and Roop, 1999; Valentin-Hansen et al., 2004). The pathogen antioxidant SodC, a Cu/Zn superoxide dismutase equip *Brucella* with the proper tools to counteract reactive oxygen species (ROS) (Gee et al., 2005). Gee et al, presented evidence supporting Hfq-dependent optimal stationary-phase *sodC* expression in *B. abortus* and showed decreased SodC production in the *hfq* mutant Hfq3 (Gee et al., 2005). Additionally, there are two other known defense mechanisms, the production of nitric oxide reductase to detoxify NO within macrophages and overexpressed by the bacterium early during infection and AhpC, a peroxiredoxin that scavenges endogenous ROS generated by *Brucella* metabolism produced in defense against H_2_O_2_ -mediated toxicity (Gross et al., 1998; Haine et al., 2006; Loisel-Meyer et al., 2006; Steele et al., 2010; Poncin et al., 2019). The progressive exclusion of LAMP1 from the vacuolar membrane of BCVs (80% loss at 6 - 12 hrs post infection), allows for the capture vesicles from ER exit sites creating a subcellular compartment with neutral pH that is permissive for replication (Starr et al., 2008). Recently it has been demonstrated by electron microscopy that at any given time, some of the replicative BCVs share a continuous lumen with the ER and speculate that the snapshots most likely reflect tethering events between these compartments (Sedzicki et al., 2018).

BCV-ER vesicle fusion is carefully coordinated by translocated effectors and requires the activity of the small GTPase, Rab2a (Fugier et al., 2009). The importance of Rab2a activity on rBCV development is shown by the fact that inhibition of Rab2 expression by small interfering RNA results in LAMP1 retention on the BCV, hindering ER vesicle fusion and ultimately inhibiting replication (Fugier et al., 2009; de Barsy et al., 2011). The effector, BspB, contributes to *Brucella’s* replicative niche by interacting with the conserved oligomeric Golgi (COG) tethering complex, a major coordinator of Golgi vesicular trafficking, thus remodeling Golgi membrane traffic and redirecting Golgi-derived vesicles to the BCV, and if BspB is deleted, *Brucella* replication in macrophages is attenuated (Miller et al., 2017). Interestingly however, the intracellular growth defect of a Δ*bspB* mutant in macrophages is reversed when host Rab2a protein levels are depleted by siRNA (Miller et al., 2017), with the mutant bacteria now able to grow. The T4SS effector RicA directly interacts with Rab2a and promotes Rab2a recruitment to the BCV, but deletion of *ricA* does not impact rBCV development in macrophages (de Barsy et al., 2011). However, deletion of *ricA* in a *bspB* mutant background suppresses the growth defect of the *bspB* mutant in macrophages, indicating an epistatic relationship between the functional roles of BspB and RicA (Myeni et al., 2013; Smith et al., 2020). This epistatic interplay between these two effectors allows *Brucella* to fine tune the modulation of host cell processes to allow development of the rBCV and promote intracellular bacterial proliferation. Another effector, BspF, promotes intracellular replication within rBCVs (Borghesan et al., 2021). BspF hijacks vesicular transport between the trans-Golgi network (TGN) and the recycling endosome pathway, and this results in the accumulation of TGN-associated vesicles to the rBCV (Borghesan et al., 2021). Therefore it is clear *Brucella* targets the host vesicular pathways by using multiple translocated effector proteins to develop a replicative niche.

Maturation of the BCV into replicative organelles is dependent upon BCV acidification and interaction with late endosomes/lysosomes allowing for biogenesis of the ER-derived replicative BCV (**Figure 1**) (Starr et al., 2008). The O-side chain of smooth LPS plays an important role in initiating and maintaining the early stages of development of the BCV while the T4SS secreted effectors are responsible for maintaining the interactions of the BCV with the ER to allow remodeling of the BCV into a replicative niche (Roop et al., 2004). Following replication, a subpopulation of *Brucella* traffic to a new subcellular compartment resembling an autophagosome that may aid in spreading to neighboring cells for a second round of infection (Starr et al., 2012). More experiments are needed to determine the intermediate steps of BCV trafficking and maturation. For example, in addition to BCV trafficking, microtubule manipulation by either depolymerization or polymerization events interfere with BCV maturation and replication of *B. abortus* (Alves-Silva et al., 2017). The effector, TcpB (also known as BtpA), possesses a TIR domain and localizes to microtubules, and in turn increases nucleation and polymerization of microtubules and acts as a stabilization factor for microtubules (Radhakrishnan et al., 2011; Alves-Silva et al., 2017). It is possible other effectors contribute to this process, to generate the rBCV. Additionally, further analysis with markers of different endosomal, Golgi- and ER-derived compartments are necessary to further characterize the trafficking of the *Brucella*-containing vacuole along the endosomal pathway and its ER-mediated remodeling (Piersanti et al., 2015). For further reading on the survival cycle in host cells by the facultative intracellular bacteria, *Brucella*, please refer to the recent review by Jiao, et al. (Jiao et al., 2021).

## Interaction of *Anaplasma-*Containing Vacuoles With Endocytic and ER-to-Golgi Vesicle Traffic

*Anaplasma* spp. are Gram-negative obligate, ixodid tick-transfected intracellular bacteria that infect neutrophils and endothelial cells of vertebrate hosts (Alberdi et al., 2016). Within the tick vector, *Anaplasma* spp. enter the tick midgut epithelium. After initial replication in tick gut cells, *Anaplasma* reach the tick salivary glands where a second round of replication occurs. Following second replication, *Anaplasma* migrate to the saliva allowing bacterial transmission to the next vertebrate host (Ueti et al., 2009). *Anaplasma* is also transmitted by biting flies or blood-contaminated fomites including needles, tattooing instruments, or nose tongs (Kocan et al., 2010; Scherler et al., 2018). Of all the ixodid tick-transmitted bacteria, *Anaplasma marginale* and *Anaplasma phagocytophilum* pose major veterinary and public health significance. In humans, the species *A. phagocytophilum* is the causative agent of human granulocytic anaplasmosis, with symptoms including high fever and leukopenia implicating both granulocytes and lymphocytes (Woldehiwet, 2006; Scherler et al., 2018). In a small number of human infections (<3%), patients can experience acute respiratory distress syndrome that can lead to death in 1% of total cases (Bakken and Dumler, 2015; Scherler et al., 2018). During infection in cattle and other ruminants, *Anaplasma marginale* replicates in erythrocytes leading to bovine anaplasmosis principally and this infection persists up to 7 weeks ultimately resulting in death (Kocan et al., 2010). In contrast to infection of *A. marginale, A. phagocytophilum* does not replicate in erythrocytes but within the vacuoles of neutrophils (Stuen et al., 2013; Scherler et al., 2018).

As an obligate intracellular pathogen, *A. phagocytophilum* has evolved the ability to inhibit host cell apoptosis through the extrinsic (the death receptor pathway) or the intrinsic pathway (the mitochondrial pathway) (Ge et al., 2005; Ge and Rikihisa, 2006; Elmore, 2007; Niu et al., 2010; Niu and Rikihisa, 2013; Alberdi et al., 2016; de la Fuente et al., 2016). The *Anaplasma* developmental cycle exhibits two distinct forms, the noninfectious replicative form, termed reticulate cell. This is followed by the infectious “dense-cored cell” form, forming microcolonies called morulae (**Figure 1**) (Troese et al., 2011; Alberdi et al., 2016). *Anaplasma* spp. have evolved diverse strategies to persistently facilitate the transmission of the pathogen between ticks and host species. Immunodominant outer membrane proteins (MSPs) present in the *Anaplasmataceae* family generate new antigenic variants in order to evade host immune response (Scherler et al., 2018). Additionally, members of *Anaplasma* genus show no evidence of peptidoglycan (PG) layer or lipopolysaccharide (LPS) biosynthesis, thus allowing *Anaplasma* to infect host cells without activating the innate immune response (Lin and Rikihisa, 2003a; Scherler et al., 2018).

Similar to *Chlamydia*, *A. phagocytophilum* intercepts host cholesterol for intracellular growth and survival. *Anaplasma* also uses cholesterol to enter host cells through caveolae or lipid rafts (Toledo and Benach, 2015). Throughout infection, the phagosome membrane maintains caveolin-1 indicating that there is a need for continuing acquisition of cholesterol (Lin and Rikihisa, 2003b). To acquire cholesterol to enhance its growth, *A. phagocytophilum* hijacks the host low-density lipoprotein (LDL) uptake pathway to obtain cholesterol from outside the host cell, rather than relying on *de novo* production of this lipid by the host cell, to bring cholesterol to its phagosome (Xiong et al., 2009). Infection by *A. phagocytophilum* increases transcription and stabilization of the host LDL receptor mRNA and also expression of the protein in host cells (Xiong et al., 2009). Additionally, the Niemann-Pick type C1 (NPC1) protein, which plays a key role in intracellular cholesterol transport, and the lipid raft protein, flotillin, are hijacked by *A. phagocytophilum* to transport cholesterol to the *A. phagocytophilum* vacuole (Huang et al., 2021), though the mechanism is not yet known. The acquisition of cholesterol by upregulating the expression of low-density lipoprotein receptors is a unique evolutionary adaptation of *A. phagocytophilum*, occupying a distinctive niche.

Alternatively, the *A. marginale*-containing vacuoles have shown to accumulate and retain the early endosomal compartment marker, Rab5, the recycling endosomal compartment markers Rab4 and Rab11, and the late endosomal compartment marker Rab7 (**Figure 1**) (Magunda et al., 2016). The association of the *A. marginale*-containing vacuoles with the ER and Golgi apparatus during early in infection and the maintained association throughout the course of infection, allows for nutrient acquisition required to establish this vacuolar replicative niche (Magunda et al., 2016). *A. marginale’s* ability to inhibit apoptosis, maintain association with the ER and Golgi apparatus during infection, and acquire cholesterol by upregulating low-density receptors allows the bacteria to continue to occupy this distinctive niche. The mechanisms utilized by *Anaplasma* for modulation of host phagosome biogenesis and to adapt to the maintain intra-vacuolar microenvironment are still to be deciphered. However, an *Anaplasma*-derived protein, APH_0032, was found to localize to the *A. phagocytophilum*-occupied vacuolar membrane (AVM) during infection of human myeloid cells, human microvascular endothelial cells, and murine neutrophils and might play a role in its phagosome biogenesis (Huang et al., 2010). Additionally, a known T4SS effector of *A. phagocytophilum, Ats-1*, manipulates the host autophagy degradation pathway by binding directly to Beclin 1, a central regulator of autophagy (Niu et al., 2012). The progress in our understanding of *Anaplasma*-host interactions is hampered by the obligate nature of the pathogen, the lack of an efficient genetic system, and the difficultly in bridging the similarities and/or differences between two tick-transmitted pathogens of *Anaplasmataceae* family.

## The Lysosomal-Like *Coxiella*-Containing Vacuole

*Coxiella burnetii* is an intracellular pathogen that initially targets alveolar macrophages, systematically manifesting as a severe flu-like illness known as Q fever (Miller et al., 2019). *Coxiella* is the only documented bacterial pathogen known to survive and proliferate within a phagolysosome. However, in specific pathological conditions, such as Crohn’s disease, adherent-invasive *Escherichia coli* replicates in phagolysosomes within macrophages (Bringer et al., 2006). The *Coxiella*-containing vacuole (CCV) undergoes endocytic trafficking within the endosomal-lysosomal pathway to mature into what is considered a bactericidal acidic phagolysosome, which contains lysosomal hydrolases that degrade most macromolecules and most pathogens (Baxt et al., 2013; Miller et al., 2019; Newton et al., 2020). Because *C. burnetii* utilizes host machinery for uptake and travels along the default endosomal-lysosomal pathway, the bacteria do not require a functional Dot/Icm T4SS system until the bacteria have established residence in an acidified lysosome-derived vacuole (Newton and Roy, 2011). The Dot/Icm system with its > 60 translocated effector proteins identified to date is crucial for replication and virulence of *C. burnetii* (Newton and Roy, 2011; Pechstein et al., 2020). This is supported by the observations that *C. burnetii* mutants defective in the Dot/Icm translocation apparatus travel to a lysosome-like vacuole, similar to the wild type strain, but yet are incapable of intracellular replication (Newton et al., 2013).

This acidic environment of the CCV triggers the delivery of the translocated T4SS system effectors into the host cytosol that likely modulate the intracellular environment to provide specific nutritional requirements conducive to its replicative niche (Newton and Roy, 2011). Studies have shown that siRNA silencing of the host Rab5 or Rab7 GTPases, or blocking acidification of the CCV, results in a significant reduction in effector translocation by *C. burnetii* (Newton et al., 2013; Newton et al., 2016; Newton et al., 2020). For example, the translocated effector AnkF, interacts with the host cytoskeletal component, vimentin, but this interaction appears to be important for establishing the CCV to become a replicative compartment, rather than directing the intracellular trafficking of the *Coxiella* vacuole (Pechstein et al., 2020). The injected serine/threonine protein kinase, CstK, influences the development of the acidified CCV through interacting with the host protein TBC1D5, a Rab7 GTPase-activating protein (Martinez et al., 2020). Hijacking TBC1D5 activity appears to be important for intracellular replication of *Coxiella* within the acidified CCV (Martinez et al., 2020). Additionally, at least two effectors, CvpF and CvpB manipulate autophagy to modify the acidified CCV into a more replicative compartment. CvpF interacts with the host Rab26, resulting in recruitment of MAP1LC3B/LC3B to the CCV (Siadous et al., 2021), a marker of autophagosomes. CvpB binds to phosphatidylinositol 3-phosphate to enhance association of the autophagosomal components to the CCV (Martinez et al., 2016). Another effector, AnkG plays a key role in anti-apoptosis, which is distinct from maturation of the CCV, though it is important in progression of disease in a *Galleria mellonella* infection model (Schafer et al., 2020). NopA is injected into host cells and impacts the innate immune response to *Coxiella* infection, but does not impact trafficking of this pathogen to a replicative CCV (Burette et al., 2020). Thus, the translocated effector proteins enable proliferation of *C. burnetii* in a spacious CCV but may not be required for progression of the CCV through the endocytic pathway (Newton et al., 2013).

Although all other intra-vacuolar pathogens have evolved and adapted to evade or subvert the endosomal/lysosomal pathway, the CCV becomes acidified, and bacteria replicate within this acidified phagolysosome (Miller et al., 2019). It is likely that there is an evolutionary advantage for delaying effector translocation until the CCV has matured into a lysosome-like vacuole. *Coxiella* remains unique as the main intra-vacuolar bacterial pathogen that proliferates within a phagolysosome. Recent technologies of *in vivo* genetic studies on obligate intracellular bacteria should move the field forward (Bekebrede et al., 2020).

## Conclusions

The main goal of default phagosome formation and maturation through a series of fission and fusion events with early endosomes, late endosomes and lysosomes is to eliminate foreign pathogens and apoptotic cells. However, despite the common objective of avoiding lysosomal fusion, intracellular bacterial pathogens including *Salmonella*, *Mycobacteria, Legionella, Chlamydia, Brucella, Coxiella*, and *Anaplasma* have evolved a diverse range of different strategies to evade or subvert the default endosomal-lysosomal pathway, generating a favorable intracellular environment for survival and replication. *Coxiella* is unique as its vacuole matures through the default endosomal-lysosomal pathway and promotes fusogenic events with lysosomes to create a lysosome-like vacuole that enables proliferation. Similar to *Coxiella*, the *Salmonella* containing vacuole has evolved to accumulate some late endosome-lysosomes markers and multiple mechanisms to allow for its adaptation and survival within an idiosyncratic acidified late endosome-like vacuole. *Mycobacteria* and *Anaplasma* retain early endosomal compartment markers halting phagosomal maturation by secreting various effector proteins that alters host signaling pathways, generating an early endosome-like replicative niche. *Legionella* also modulates host signaling pathways by way of remodeling the LCV into an ER-derived vacuole which diverts traffic away from the endosomal-lysosomal pathway. *Chlamydia* and *Anaplasma* can divert their bacteria containing vacuoles from the endosomal-lysosomal pathways and intercept vesicles to incorporate lipids and cholesterol into their membranes. While *Legionella, Chlamydia, Brucella* and *Anaplasma* all exhibit ER vesicle fusion, this is carefully coordinated by specific secreted effectors that function with different mechanisms. The *Brucella* containing vacuole also initially fuses lysosomal compartments but then is remodeled by ER-derived vesicles to enable proliferation. Therefore, this clearly demonstrates the unique strategies evolved by diverse intracellular pathogens to overcome the host defensive strategy of phagosome/lysosomal fusion.

Understanding these idiosyncratic mechanisms has provided novel insights into the mechanisms of host-pathogen interactions and will continue to unravel new strategies for the control and prevention of infectious diseases. Furthermore, the ability of intracellular bacterial pathogens to modulate their phagosome has provided knowledge on how the default endosomal-lysosomal pathway functions in eukaryotes, which has a broad importance in the understanding of eukaryotic cell biology. The bacterial effectors involved in host cell modulation through interaction with specific host targets in these pathways are potential biotechnology tools that can be utilized to decipher cell biology. Future studies will reveal how intra-vacuolar pathogens have evolved and adapted with such idiosyncratic mechanisms employing a unique set of injected effectors to modulate the default endosomal-lysosomal and as well as the exocytic pathways and will further increase the understanding of these essential pathways in eukaryotes. While there has been a quantum leap in our knowledge of modulation of phagosome biogenesis by intra-vacuolar pathogens, the detailed biochemical and cellular processes affected remain to be deciphered for most intra-vacuolar pathogens. Therefore, future studies should be aimed at expanding the mechanisms of how bacterial effectors used by intra-vacuolar pathogens biochemically and/or physiologically modulate phagosome biogenesis. Furthermore, research has yet to elucidate the temporal and hierarchical delivery of effectors involved in phagosome biogenesis. The new era of single-cell biology of deciphering host-microbe interaction should facilitate future studies (Hayward et al., 2019; Sharma and Thaiss, 2020).

## Author Contributions

BV collected and assembled the information and drafted the article. BV and YK critically revised the article. All authors contributed to the article and approved the submitted version.

## Funding

The YK lab is supported by Public Health Service Awards R01AI120244, R01AI140195 and R21AI142727 from the NIAID and by the Commonwealth of Kentucky Research Challenge Trust Fund.

## Conflict of Interest

The authors declare that the research was conducted in the absence of any commercial or financial relationships that could be construed as a potential conflict of interest.

## Publisher’s Note

All claims expressed in this article are solely those of the authors and do not necessarily represent those of their affiliated organizations, or those of the publisher, the editors and the reviewers. Any product that may be evaluated in this article, or claim that may be made by its manufacturer, is not guaranteed or endorsed by the publisher.
